# Ambient Particulate Matter Induces In Vitro Toxicity to Intestinal Epithelial Cells without Exacerbating Acute Colitis Induced by Dextran Sodium Sulfate or 2,4,6-Trinitrobenzenesulfonic Acid

**DOI:** 10.3390/ijms25137184

**Published:** 2024-06-29

**Authors:** Candace Chang, Allen Louie, Yi Zhou, Rajat Gupta, Fengting Liang, Georgina Xanthou, Jason Ereso, Carolina Koletic, Julianne Ching Yang, Farzaneh Sedighian, Venu Lagishetty, Nerea Arias-Jayo, Abdulmalik Altuwayjiri, Ramin Tohidi, Mohamad Navab, Srinivasa Tadiparthi Reddy, Constantinos Sioutas, Tzung Hsiai, Jesus A. Araujo, Jonathan P. Jacobs

**Affiliations:** 1 Vatche and Tamar Manoukian Division of Digestive Diseases, David Geffen School of Medicine, University of California Los Angeles, Los Angeles, CA 90095, USA; candacechang328@g.ucla.edu (C.C.); zhouyisic@163.com (Y.Z.); fengting.liang@gmail.com (F.L.); gxanthou@bioacademy.gr (G.X.); jasonereso@g.ucla.edu (J.E.); ckoletic@mednet.ucla.edu (C.K.); jcyang1617@g.ucla.edu (J.C.Y.); sedighianfarzaneh@gmail.com (F.S.); vlagishetty@gmail.com (V.L.); nere.arias3@gmail.com (N.A.-J.); 2 Division of Cardiology, David Geffen School of Medicine, University of California Los Angeles, Los Angeles, CA 90095, USA; allenlouie@g.ucla.edu (A.L.); rajatgupta@ucla.edu (R.G.); mnavab@mednet.ucla.edu (M.N.); sreddy@mednet.ucla.edu (S.T.R.); thsiai@mednet.ucla.edu (T.H.); 3 Department of Environmental Health Sciences, Fielding School of Public Health, University of California Los Angeles, Los Angeles, CA 90095, USA; 4 Molecular Toxicology Interdepartmental Program, University of California Los Angeles, Los Angeles, CA 90095, USA; 5 West China Medical Center, Sichuan University, Chengdu 610017, China; 6 USC Viterbi School of Engineering, University of Southern California, Los Angeles, CA 90089, USA; a.altuwayjiri@mu.edu.sa (A.A.); tohidi@usc.edu (R.T.); sioutas@usc.edu (C.S.); 7 Department of Civil and Environmental Engineering, College of Engineering, Majmaah University, Al-Majmaah 11952, Saudi Arabia; 8 Air Quality Planning and Science Division, California Air Resources Board, 4001 Iowa Avenue, Riverside, CA 92507, USA; 9 Molecular & Medical Pharmacology, University of California Los Angeles, Los Angeles, CA 90095, USA; 10 Henry Samueli School of Engineering, University of California Los Angeles, Los Angeles, CA 90095, USA; 11 Division of Gastroenterology, Hepatology and Parenteral Nutrition, Veterans Administration Greater Los Angeles Healthcare System, Los Angeles, CA 90073, USA; 12 Goodman-Luskin Microbiome Center, University of California Los Angeles, Los Angeles, CA 90095, USA

**Keywords:** particulate matter, ultrafine particles, inhalation, colitis, inflammation

## Abstract

Inflammatory bowel disease (IBD) is an immunologically complex disorder involving genetic, microbial, and environmental risk factors. Its global burden has continued to rise since industrialization, with epidemiological studies suggesting that ambient particulate matter (PM) in air pollution could be a contributing factor. Prior animal studies have shown that oral PM_10_ exposure promotes intestinal inflammation in a genetic IBD model and that PM_2.5_ inhalation exposure can increase intestinal levels of pro-inflammatory cytokines. PM_10_ and PM_2.5_ include ultrafine particles (UFP), which have an aerodynamic diameter of <0.10 μm and biophysical and biochemical properties that promote toxicity. UFP inhalation, however, has not been previously studied in the context of murine models of IBD. Here, we demonstrated that ambient PM is toxic to cultured Caco-2 intestinal epithelial cells and examined whether UFP inhalation affected acute colitis induced by dextran sodium sulfate and 2,4,6-trinitrobenzenesulfonic acid. C57BL/6J mice were exposed to filtered air (FA) or various types of ambient PM reaerosolized in the ultrafine size range at ~300 μg/m^3^, 6 h/day, 3–5 days/week, starting 7–10 days before disease induction. No differences in weight change, clinical disease activity, or histology were observed between the PM and FA-exposed groups. In conclusion, UFP inhalation exposure did not exacerbate intestinal inflammation in acute, chemically-induced colitis models.

## 1. Introduction

Between 1990 and 2017, the incidence of inflammatory bowel disease (IBD), a debilitating condition characterized by chronic inflammation in the gastrointestinal tract, increased by 31% globally [1]. IBD encompasses two clinical diagnoses—Crohn’s disease and ulcerative colitis—with pathogenesis involving an interplay between genetic susceptibility and environmental factors such as diet. Environmental triggers may be the cause for the increasing prevalence of IBD in industrialized countries, with more than 3.6 million people in the United States affected by IBD [2]. Intriguingly, urban living has been associated with an increased risk of IBD compared to rural living in studies throughout the world [3,4]. Among the potential risk factors associated with urban living, increased air particulate matter levels have been linked to IBD diagnosis and exacerbation in epidemiological studies [5,6,7]. Long-term exposure to high concentrations of nitrogen dioxide and particulate matter (PM) has been associated with an increased risk of early-onset Crohn’s disease [5,8]. Studies have also correlated the increased density of ambient air pollution with IBD hospitalizations [5,9]. These studies suggest that air pollution may be an environmental factor contributing to the development of IBD. This would be consistent with the ability of PM to trigger inflammation in systemic tissues leading to a greater propensity for myocardial infarction [10], arthritis [11], and appendicitis [12].

PM can be categorized by size into PM10 (aerodynamic diameter <10 μm), fine particles or PM_2.5_ (<2.5 μm diameter), and ultrafine particles or UFP (aerodynamic diameter < 0.10 μm). UFP represents 85–90% of PM_2.5_ by number and more than 80% of total industrial and urban ambient particle numbers [13,14]. Detrimental health effects may increase with decreasing particle size [15]. Surface reactivity and aspect ratio, among other properties, enable greater access of UFP to peripheral airways and potential uptake into the circulation [16]. Their small size results in increased surface area relative to mass, further increasing their biological activity [17]. Cumulative evidence indicates that UFP exposure is associated with the risk of diseases affecting the pulmonary [18], central nervous [19], and cardiovascular systems [20]. This may occur by various mechanisms including increased oxidative stress and cell membrane permeability [21,22]. Particulate matter also induces higher levels of various proinflammatory cytokines. Thus, alveolar macrophages exposed to PM exhibit increased levels of IL-6, IL-1β, and GM-CSF [23], which are also found elevated in the serum of human subjects after exposure to an acute air pollution episode [23].

PM has the potential to reach the gastrointestinal tract after inhalation via mucociliary transport and systemic absorption. Particles > 6 μm have been shown to translocate via mucociliary clearance [24,25]. Smaller particles such as ultrafine particles may gain access to organs via mucociliary clearance as has been shown for larger particulates [24,26], or after dissemination via systemic circulation [27,28]. Once in the gastrointestinal tract, ambient particles have the potential to promote intestinal inflammation through effects on the intestinal epithelium, intestinal immune cells, and gut microbiota [29]. Oral ingestion of PM_10_ altered the microbiome and generated inflammatory responses in IL-10^−/−^ mice—a genetic model of spontaneous colitis—7 and 14 days after gavage [30]. Elevated inflammatory markers in IL-10^−/−^ mice were also reported after 10 and 14 weeks on a chow diet containing PM_10_ [31]. Orogastric administration of UFPs has been reported to induce intestinal inflammation in hyperlipidemic *Ldlr*^−/−^ mice [32]. A study using C57BL/6J mice showed that inhalation exposure to concentrated PM_2.5_ for three weeks resulted in increased colonic mRNA expression of TNF-α [33]. In contrast, we previously reported that mRNA levels of proinflammatory cytokines (IL-1β, IFNγ, TFNα) were similar between PM and FA-exposed mice in the jejunum and colon of *ApoE*^−/−^*, Ldlr*^−/−^, and C57BL/6J mice exposed for 10 weeks, without histological evidence of inflammation [34]. Altogether, it is possible that inhalation exposure to PM promotes inflammation in specific contexts, potentially in susceptible subjects or in the presence of other triggers of colitis which could precede or act at the same time as the PM exposures. Thus, mixed results on the induction of subclinical intestinal inflammation in mice exposed to inhaled PM but encouraging findings from oral exposure studies in the IL-10^−/−^ colitis model led us to hypothesize that exacerbation of colitis models would be an alternative approach to model the impact of inhaled PM on IBD development. This is consistent with existing paradigms of the effects of environmental exposures on IBD, which could act at varying pathophysiologic stages including exacerbation of an existing subclinical inflammatory process to induce clinical onset of disease [35]. To test our hypothesis, we assessed the effects of inhalation exposure to PM reaerosolized in the ultrafine size range on two distinct models of acute colitis, induced by dextran sodium sulfate (DSS) and 2,4,6-trinitrobenzenesulfonic acid (TNBS) (Supplementary Figure S1).

## 2. Results

### 2.1. In Vitro PM Exposure Demonstrated Toxicity in Intestinal Cells

In vitro experiments were performed to evaluate PM toxicity in the Caco-2 intestinal cell line. Four distinct batches of ambient PM were tested, collected via different collection methods, and from different geographic sources: Athens, Milan, and Los Angeles (LA, collected as either suspended [sPM] or nanosized PM [nPM] as described in the Methods). LA (sPM), Milan PM, and Athens PM were collected as PM_2.5_ while LA (nPM) was collected as UFP. Analysis of the chemical composition of various PM batches showed that the main observed differences were less organic matter in the LA (nPM) compared to the other three PMs, as well as reduced metal elements in Milan compared to the other PMs (Supplementary Table S1). Water-soluble inorganic ions were the dominant fraction of Los Angeles (sPM) (~43%), followed by organic matter (~39%) and metal elements (~18%), while Los Angeles (nPM) sample included a similar proportion of inorganic ions (44%), less organic matter (28%) and more metal elements (27%). Milan samples exhibited high levels of inorganic ions (45%) and organic matter (46%), while Athens showed high levels of organic matter (44%) and moderate loading of inorganic ions and metal elements (36% and 20%, respectively).

**Figure 1:**
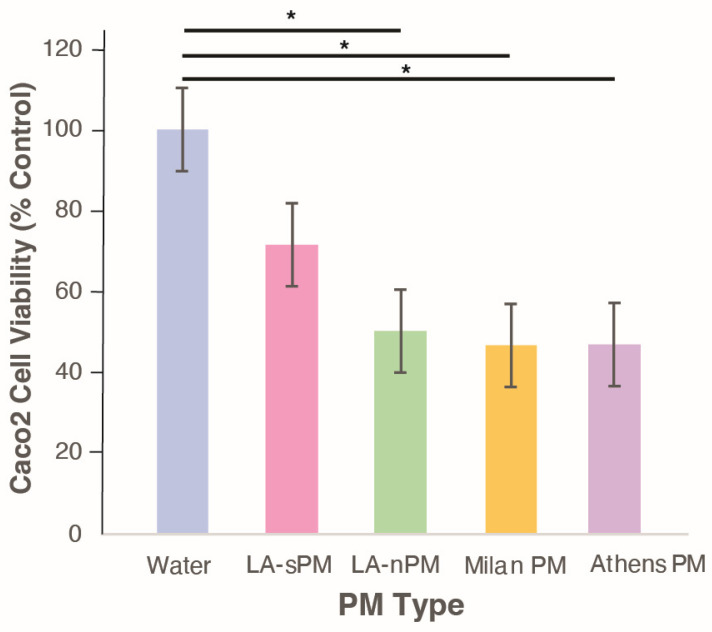
Diverse PM sources induced toxicity to cultured Caco-2 intestinal epithelial cells. In vitro MTT assay for cell viability and cytotoxicity. Ultrapure milli-Q water was used as a negative control. Each PM sample was diluted to 25 μg/mL in the treatment media. * *p* < 0.05. Abbreviations: Los Angeles suspended particulate matter (LA-sPM), Los Angeles nano-particulate matter (LA-nPM).

Caco-2 cells were seeded in 24-well plates and exposed to PM samples, each diluted to a concentration of 25 μg/mL in the treatment media for 24 h. We have previously shown that this PM concentration can increase the permeability of Caco-2 cells to Streptavidin-HRP [32]. Cell viability was assessed by mitochondrial dehydrogenase conversion of MTT reagent (3-(4,5-dimethylthiazol-2-yl)-2,5-diphenyl-2H-tetrazolium bromide) to purple formazan. In-vitro experiments showed all PMs induced biological toxicity in intestinal cells, with LA (nPM), Milan PM, and Athens PM significantly (*p* < 0.05) decreasing cell viability as compared to the control (Figure 1). There were no significant differences in cell viability between cells exposed to these three PM types despite differences in particle size and the source of origin, indicating that PM was toxic in this cell line across a range of various chemical compositions.

### 2.2. Inhalation Exposure to PM from Four Distinct Sources Did Not Exacerbate DSS Colitis Severity

**Figure 2:**
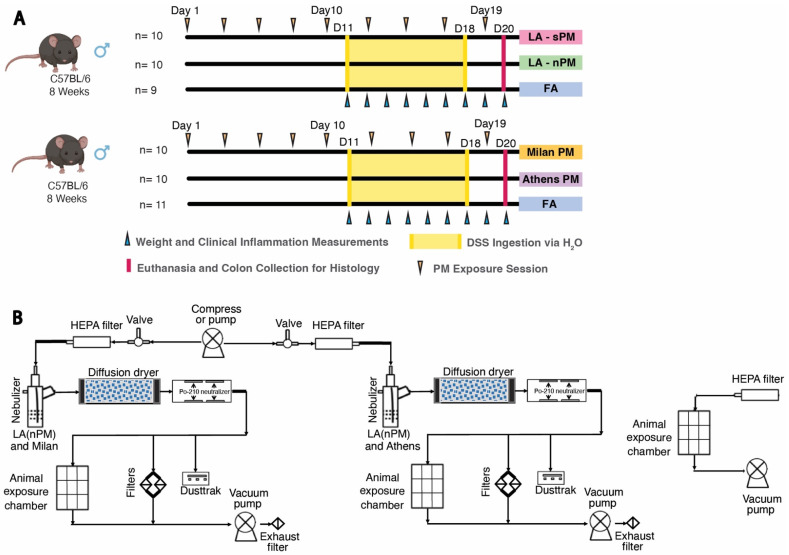
PM inhalation exposure during acute colitis induced by dextran sodium sulfate (DSS). (**A**) Experimental design. C57BL/6J mice were exposed to PM reaerosolized in the ultrafine PM size range 3 times per week over 3 weeks, for a total of 9 sessions. Daily weight measurements and fecal pellet collections were performed from Day 11–20, and fecal pellets were collected to assess stool consistency and blood. Mice were euthanized on Day 20 for colon tissue collection. Schematics were created with Biorender. (**B**) Exposure apparatus. PM reaerosolization chambers (LEFT), Filtered air exposure chambers (RIGHT). Abbreviations: Filtered Air (FA), Dextran Sodium Sulfate (DSS), particulate matter (PM), Los Angeles suspended particulate matter (LA-sPM), Los Angeles nano-particulate matter (LA-nPM), Day 11, 18, 20 (D11, D18, D20).

**Figure 3:**
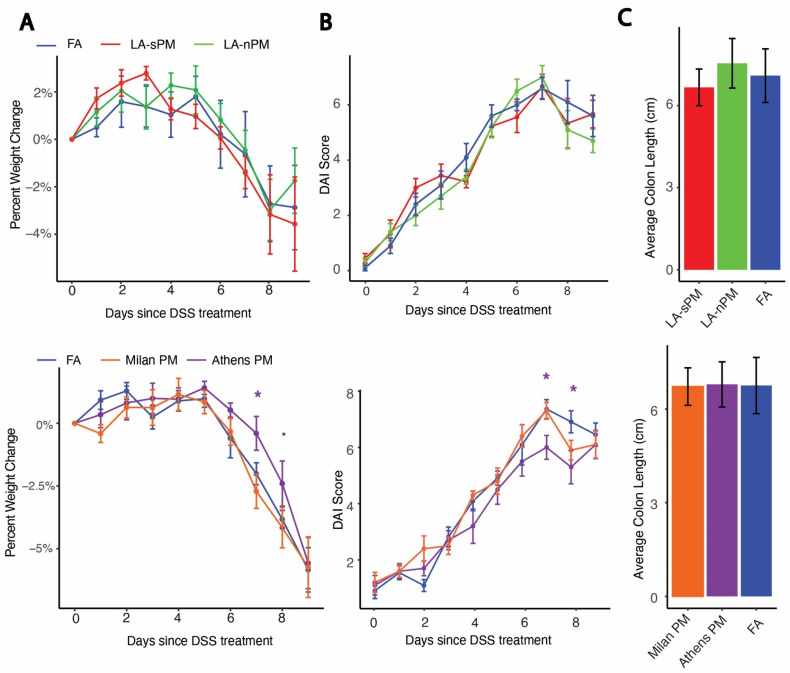
PM inhalation exposure did not increase the clinical severity of DSS colitis. (**A**) Percent weight loss, (**B**) disease activity index (DAI), and (**C**) colon length measured in centimeters of DSS-treated mice exposed to UFP or FA in the two experimental cohorts. (■ *p* < 0.1, * *p* < 0.05). Abbreviations: Dextran Sodium Sulfate (DSS), Particulate Matter (PM), Los Angeles suspended particulate matter (LA-sPM), Los Angeles nano-particulate matter (LA-nPM), Filtered Air (FA), Disease Activity Index (DAI).

**Figure 4:**
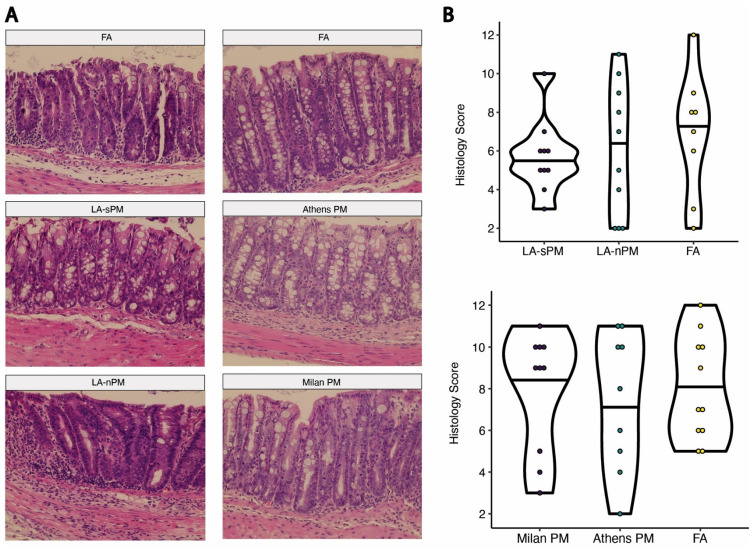
No effect of PM exposure on histological severity of DSS colitis. (**A**) Representative H&E stained colon tissues (40× magnification) from each exposure group. (**B**) Violin plots of histological scores. Abbreviations: Particulate Matter (PM), Los Angeles suspended particulate matter (LA-sPM), Los Angeles nano-particulate matter (LA-nPM), Filtered Air (FA).

We used a model of acute colitis induced by DSS to test whether inhalation exposure to the four batches of PM tested above acutely exacerbates colitis. DSS chemically induces colitis by exerting toxicity on the epithelial cells of the basal crypts in the colon [36]. Colitis severity after acute DSS exposure model is driven by several factors including loss of epithelial barrier integrity, increased innate immune responses, and decreased tissue repair mechanisms [37]. Subacute PM inhalation exposure was modeled by exposing C57BL/6J mice to the four different batches of ambient PM, reaerosolized in the ultrafine size range at 300 μg/m^3^ vs. filtered air (FA), in 6-h exposures sessions, 3 days/week over 20 days, for a total of 9 sessions. Two cohorts were used, each comparing two PM samples vs. FA (Figure 2A). On day 11 of the PM exposure, the drinking water was replaced with 2% DSS and provided ad libitum to induce acute colitis. DSS exposure occurred concurrently with PM inhalation exposure until the end of 18 days from the first PM exposure, when DSS water was replaced with regular water. Mice were euthanized, and tissues were collected at day 20 from the first PM exposure. Colitis severity after initiation of DSS treatment was assessed by weight change and Disease Activity Index (DAI), which was calculated by adding together subscales for percent weight change, stool consistency, and stool blood (fecal occult blood test) [38]. There were no significant differences in percent weight change between the PM and FA groups with the exception of the Athens PM group, which showed reduced weight loss compared to FA on day 7 (*p* = 0.035) and to a lesser extent on day 8 (*p* = 0.054) (Figure 3A). Similarly, there were no significant differences in DAI between the PM and FA groups other than the Athens PM group, which demonstrated reduced DAI scores compared to FA on days 7 (*p* = 0.035) and 8 (*p* = 0.015) (Figure 3B). Colon length was measured as a morphological assessment of inflammation, and no differences between PM and FA were observed (Figure 3C). We further performed double-blind histological scoring of the colon, which did not demonstrate differences in mucosal architecture or extent of inflammation between the PM and FA groups (Figure 4).

### 2.3. TNBS Colitis Severity Is Unaffected by PM Inhalation Exposure

**Figure 5:**
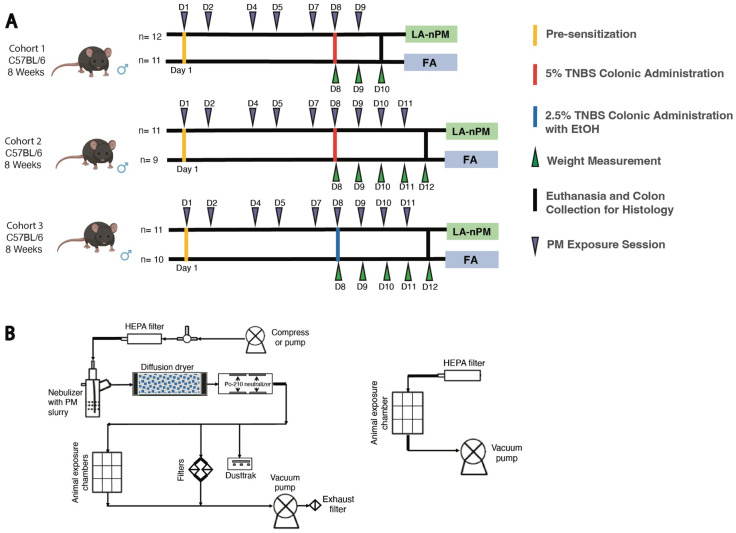
TNBS experimental design and PM characterization. (**A**) Schematic of PM and TNBS exposure in 3 cohorts. Figure 5A was created with Biorender. (**B**) Exposure apparatus. PM reaerosolization chamber (LEFT). Filtered air exposure chamber (RIGHT). Abbreviations: 2,4,6-trinitrobenzenesulfonic acid (TNBS), Particulate Matter (PM), Los Angeles nano-particulate matter (LA-nPM), Ethanol Alcohol (EtOH), Filtered Air (FA). Day 1,2,4, etc. (D1, D2, D4, etc.).

**Figure 6:**
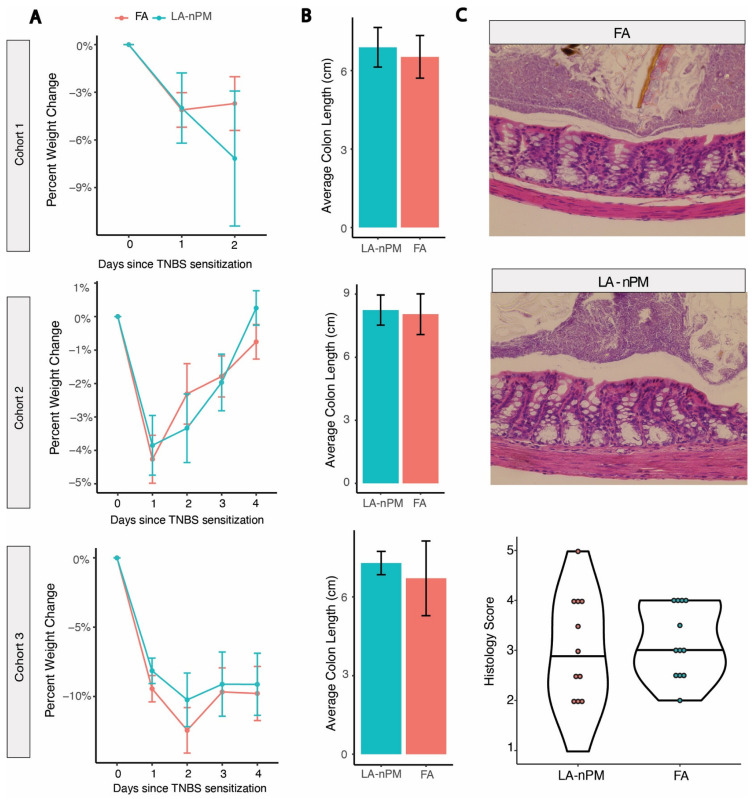
No effect of PM exposure on TNBS colitis severity. (**A**) Daily average percent weight change of the three TNBS cohorts. (**B**) Colon length at the time of euthanasia for the three cohorts. (**C**) Representative colon histology images (40× magnification) for the PM and FA groups from TNBS cohort 1 and a violin plot showing histology scores. Abbreviations: 2,4,6-trinitrobenzenesulfonic acid (TNBS), Los Angeles nano-particulate matter (LA-nPM).

To further investigate the effect of PM inhalation on acute colitis, we utilized a second model of acute colitis driven by distinct immunological mechanisms. The TNBS model functions by inducing a Th1 response to an antigen (TNBS) administered into the colon after initial sensitization by skin exposure [39]. It is believed to be most relevant to Crohn’s disease based on its histological features and dependence on NOD2 (nucleotide-binding oligomerization domain 2), which has been strongly implicated in the pathogenesis of Crohn’s [40]. Deletion of NOD2 decreased the severity of TNBS-colitis, an effect that is rescued by lymphocyte-predominant cells by adoptive transfer from ethanol-treated mice [41]. Ethanol is typically added to TNBS protocols to disturb the epithelial layer, allowing TNBS to interact with the intestinal wall to increase severity. Since there were no differences found between animals exposed to the different types of PM used in our DSS experiments, we decided to focus on a single particle type, the only PM batch collected as UFP, LA (nPM) (Supplementary Table S2). The TNBS experiments were divided into three cohorts with different induction regimens or experiment durations. Two experiments were performed with moderate TNBS severity (intrarectal TNBS administration without ethanol), one in which mice were sacrificed after 2 days for histology and one in which mice were followed for 4 days until clinical resolution (Figure 5A). A third experiment was conducted with greater disease severity due to the introduction of ethanol with rectal TNBS administration. Mice were exposed for 6-h exposure sessions to filtered air (FA) or ambient PM reaerosolized in the ultrafine size range at 300 μg/m^3^, every 1–2 days for a total of 7 sessions over 10 days for cohort 1 and 9 sessions over 12 days for cohorts 2 and 3. The PM exposure system in the TNBS was similar to the setup used for the DSS experiment, except that it uses just one exposure line containing LA (nPM) (Figure 2B). No differences were observed in the average percent weight loss between the PM and FA groups in any of the experiments (Figure 6A). There were also no significant differences in colon length between the two groups in all three experiments (Figure 6B). Mean histological scores obtained from cohort 1 (sacrificed at peak disease severity on day 2) showed no differences in histological measures of inflammation between the PM and FA groups (*p* = 0.56) (Figure 6C).

## 3. Discussion

This study is the first to report on the in vitro toxicity of PM to intestinal epithelial cells and the lack of effects of PM inhalation on acute models of chemically induced colitis. PM inhalation exposures represent a physiological route of administration in contrast to prior PM colitis experiments, which have used oral administration. Our rigorous, controlled exposures are a suitable representation of exposure to real air pollution. Our study utilized two distinct models of acute colitis with differing disease mechanisms to capture varying aspects of potential IBD pathophysiology. The DSS model induces IBD primarily via intestinal epithelial injury, disrupting the barrier and exposing mucosal immune cells to luminal antigens to incite an inflammatory response. The TNBS model promotes IBD via a T-cell-driven process. We observed that exposure to ultrafine PM by inhalation did not exacerbate inflammation in either acute DSS or TNBS colitis, despite the in vitro toxicity of the various PM sources to cultured intestinal epithelial cells. This suggests that in vivo toxicity by inhalation may require different exposures than evaluated in this study that involve higher doses, different particle sizes, or longer duration, as well as possibly models with different mechanisms of colitis induction.

Multiple PM sources were used for the DSS model to assess the possibility of varying biological effects of PM based on their source or method of collection. PMs from Milan and Athens were collected on filters and extracted in liquid, while PMs from LA were collected both from aerosols into liquid (LA-sPM) and from filters and extracted in liquid (LA-nPM). Comparisons on the toxicological characteristics and constituents of particles collected from these two methods have been previously published, and it was found that sPMs showed a nonsignificant increase in the capture of semi-volatile organic compounds [42]. Collections conducted during different seasons caused greater variation in n-alkanes than between PMs from the different collection methods. Similar oxidative potential was observed between the PMs collected on ambient filters and in liquid suspension, demonstrating that both methods are representative of ambient PM. The dominant source for Athens PM has been reported to be secondary aerosols [43], formed by gas-phase precursors emitted mainly by traffic and biomass combustion that react with ozone and hydroxyl radicals to form lower-volatility products that partition in the particle phase. The dominant source for Milan PM was biomass burning for residential heating [44], while vehicular emissions were the dominant source of LA PMs [45]. Although the atmospheric PM across the three cities originated from a variety of sources, the PM collected in each city represents the impact of the dominant emission sources in the area. Differences in where PMs were collected have previously been associated with variations in the effects of PM on immune cell activation, with PM derived from vehicular emissions and industrial activity inducing greater injury than secondary organic aerosols [43,46,47]. While all PM batches induced toxicity in Caco-2 cells in vitro, none of them exacerbated acute colitis in two different mouse models following inhalation in vivo. A limitation of the study is that a 2D cell culture model was used. 3D cell culture models would improve the in vivo relevance of cell culture findings. Another limitation is that only male mice were used, so we cannot rule out that the effects of PM exposure would have been seen in females. Since PM was reaerosolized in the ultrafine size range at similar concentrations, differences in the aerosols were based on their different chemical compositions. This allowed us to determine that the lack of effect on intestinal inflammation occurred in spite of testing PMs with large differences in chemical composition.

It is possible that higher doses during inhalation exposure may be required to increase colitis severity, and due to resource limitations, our study used one dosage rather than a range of PM doses. However, the exposures were a reasonable approximation of human exposure. The PM dosage employed in the exposure aerosols was 300–350 μg/m^3^. As we discussed in greater detail in earlier publications [48], the corresponding human PM exposure level resulting in the same dose per kg of body weight and for the same exposure period as a percent of the species’ lifetime is ~35 μg/m^3^, which is quite typical of human PM exposures in Los Angeles and other urban areas of the US [1]. Alternatively, the lack of effect of inhaled UFP may reflect the differential transport of larger-sized PM compared to ultrafine PM to the gastrointestinal tract by mucociliary transport, which has been suggested to primarily act upon particles above 6 μm [29]. Experiments testing PM_2.5_ and PM_10_ inhalation exposures at equivalent doses as used for UFP would be required to further investigate this possibility. In addition, a longer duration of PM exposure prior to DSS or TNBS administration may be required to significantly modulate inflammation in these models.

Previous PM inhalation studies found that urban coarse PM caused inflammatory effects [49,50], and in the context of colitis models, there are 2 existing studies on ingested PM_10_ effects on IL-10^−/−^ colitis, which both reported increased inflammation [30,31]. Kish et al. (2013) showed increased histological damage and proinflammatory cytokine expression following gavage of PM_10_, while Salim et al. (2014) reported increases in IFN-γ and TNFα, as well as decreases in IL-17 and IL-1β in IL-10^−/−^ fed PM_10_. The different outcomes of these prior colitis studies compared to ours may reflect pathophysiological differences between IL-10^−/−^ colitis and the two chemically-induced colitis models, the distinct routes of PM exposure (oral vs. inhalation), and different particle source and size distribution. Interestingly, we recently reported that 10-week PM inhalation exposure in hyperlipidemic and normolipidemic mice did not induce changes in proinflammatory cytokines in the intestine [34]. This suggests that the inflammatory effects of chronic PM inhalation may be more visible in genetic models predisposed to IBD, such as IL-10^−/−^ mice. Future studies are warranted with chronic models of colitis and chronic PM inhalation exposure to further investigate the impact of air pollution inhalation on IBD.

## 4. Materials and Methods

### 4.1. Animal Subjects

124 C57BL/6J male mice, 4 to 7 weeks old, were purchased from Jackson Laboratories and acclimated in our facilities until reaching 8 weeks of age. Mice were fed an autoclaved chow diet ad libitum except during exposures. Mice were housed in autoclaved shoe-box-type cages with cornhusk bedding. Our research protocol (ARC-2019-026) was conducted in compliance with the Animal Research Committee and Institutional Animal Care and Use Committee (IACUC) at the University of California, Los Angeles (UCLA) and performed in coordination with the Division of Laboratory Animal Medicine (DLAM) at UCLA.

### 4.2. Collection of Particulate Matter

Four different batches of PM were collected between August 2020 and January 2021 from different geographic locations, employing different collection methods. Thus, our study includes PM_2.5_ collected from Milan and Athens onto Teflon filters (Milan PM and Athens PM, respectively), PM_2.5_ collected from Los Angeles into liquid aqueous slurries resulting in suspended PM (LA-sPM), and UFP from Los Angeles through filters resulting in PM in the nano-size range (LA-nPM). Milan PM, Athens PM, and LA-nPM were collected on PTFE membrane filters (20 × 25 cm, 3.0 μm pore size, PALL Life Sciences, Port Washington, NY, USA) using a high-volume sampler (with a rate of 400 lpm) connected to a PM pre-impactor for separation [51].

LA—sPM was collected directly as aqueous slurries via the versatile aerosol concentration enrichment system (VACES)/aerosol-into-liquid-collector tandem technology. Two collection campaigns were performed (Table 1). Collection of PMs for the DSS experiments and the first TNBS cohort occurred between December 2020 and January 2021 (Table 1), while PM samples used in the second and third TNBS experiments were LA—nPM collected between December 2020 and February 2021. Each filter was divided into 32 pieces and extracted in Milli-Q water using 1 h of sonication. The amount of extracted PM via sonication was obtained by subtracting the pre-extraction from the post-extraction weights of the filters using a high-precision (±0.001 mg) microbalance (MT5, Mettler Toledo Inc., Columbus, OH, USA). Further details regarding PM collection and extraction have been reported by us [42,52].

### 4.3. In Vitro Experiments

Caco-2 cells, a human colon adenocarcinoma cell line, were cultured in Dulbecco’s minimum essential medium (DMEM) with 20% filtered, heat-inactivated fetal bovine serum (FBS), 1% MEM non-essential amino acids, and 1% Penicillin-Streptomycin at 37 °C, 5% CO_2_, and 100% relative humidity. Cells were seeded in growth media in a 96-well plate for 24 h before PM treatment, which was performed with 3 biological replicates per PM sample. All PM samples were prepared by sonication in a water bath for 10 min and diluted to a concentration of 25 μg/mL in the treatment media (DMEM). For LA (nPM), Milan, and Athens PMs, the stock concentration was 200 μg/mL (Table 2); a blank of 125 μL MilliQ water and 875 μL of media was used to compare cell viability. For LA (sPM), the stock concentration was 65 μg/mL, and a corresponding blank of 384.61 μL MilliQ water and 615.39 μL media was used. At the time of seeding, there were 1.51 × 10^6^ cells/mL and the live count was 7.39 × 10^5^ (49% live cells). Cell viability was assessed by the MTT assay; average absorbance at 560 nm was calculated between three experimental wells in triplicates. Cell viability for each PM sample type was calculated using this formula: (average absorbance of each PM treatment/average absorbance of the experimental blank) × 100.

### 4.4. DSS PM Inhalation Exposures

Inhalation exposures were conducted at the Air Pollution Inhalation Exposure Facility (APIEF), located within the animal vivarium (5V) in the Center for Health Sciences building at UCLA [53]. Following at least 1 week of acclimatation, the C57BL/6J were 8 weeks of age at the start of the experiment. The exposure protocol consisted of 6-h exposure sessions to filtered air (FA) or ambient ultrafine PM at 300 μg/m^3^, 3 days/week over 20 days, for a total of 9 sessions (Figure 1). Exposed mice were placed in exposure chambers that housed up to 22 mice/chamber. A compressor pump built at the University of Southern California (USC) Viterbi School of Engineering pushed HEPA-filtered air into a Hope nebulizer (B&B Medical Technologies, USA) to reaerosolize the different PM-containing solutions into the ultrafine size range [52] as previously described [34] (Figure 2B). Two parallel exposure lines were running for LA—nPM and LA—sPM in our first exposure campaign, and Milan and Athens PM exposures were conducted in a subsequent campaign following the first. The reaerosolized PM was drawn through a silica gel diffusion dryer (Model 3620, TSI Inc., Shoreview, MN, USA) followed by Po-210 neutralizers (Model 2U500, NRD Inc., Grand Island, NY, USA) to remove the excess water content and electrical charges of the particles, respectively. The air stream entered the animal exposure chamber with a flow rate of 2.5 lpm for in vivo inhalation exposure assessments. In parallel, the reaerosolized particles were collected on PTFE (Teflon) and Quartz (37-mm, Pall Life Sciences, 2-μm pore, Ann Arbor, MI, USA) filters to investigate the chemical characterization of particles in the system. Furthermore, variations in PM mass concentration were measured by TSI DustTrak during operation, where the average PM concentration was set at about 300 μg/m^3^. An adjacent control chamber was used in exposure experiments in which ambient air was passed through a HEPA filter and drawn into the chamber via a vacuum pump. On day 11 of the PM exposure, the drinking water was replaced with 2% DSS in de-ionized water and provided ad libitum to induce acute colitis. DSS exposure occurred concurrently with UFP inhalation exposure until the end of 18 days from the first exposure. DSS water was then replaced with regular water for 2 days before euthanasia on day 20 [54]. Weight, stool consistency, and rectal bleeding were monitored daily after the initiation of DSS to assess colitis severity. The mice were euthanized under isoflurane anesthesia 18 h after the last exposure. Colon tissue was harvested after euthanasia, as previously described [55]. Colon length, excluding the cecum, was measured in centimeters. Colon length was assessed after euthanasia as a morphological assessment of intestinal inflammation, with shorter colon length indicating greater colitis severity.

### 4.5. DSS PM Characterization

The particles collected on filters and slurries were chemically analyzed for their constituents, including carbonaceous content, water-soluble inorganic ions, and metal elements, by the Wisconsin State Laboratory of Hygiene (WSLH) [52].

### 4.6. TNBS PM Exposures

TNBS experiments included three cohorts of 8-week-old male C57BL/6J mice exposed to LA-nPM vs. FA, conducted at the same location, using a similar exposure apparatus set-up, and PM concentrations as described for DSS. Cohort 1 was exposed for 7 sessions over 10 days, while cohorts 2 and 3 were exposed for 9 sessions over 12 days. All three mouse cohorts were pre-sensitized to TNBS in the morning before their first PM exposure session. This involved shaving the skin on the back of the neck, followed by administration of the pre-sensitization solution containing 4 parts acetone/olive oil and 1 part 5% TNBS solution for a final concentration of 1% TNBS as described in the literature [56]. Cohort 1 received intrarectal administration of 150 μL of a 5% TNBS solution under anesthesia by isoflurane vapor on Day 8 of PM exposure. Cohort 2 also received the same volume of a 5% TNBS solution. Cohort 3 received intrarectal administration of 150 μL of a 2.5% TNBS solution (1 volume of 5% TNBS mixed in water with 1 volume of absolute ethanol).

### 4.7. Clinical Measures of Inflammation Analysis

Daily body weights were measured from the onset of the DSS or TNBS regimen (day 11 and day 8, respectively). Fecal pellets were also collected daily from DSS mice starting from day 11. Percent weight change on each day was calculated using this formula: % weight change = 100 × (baseline weight × 100)/(baseline weight), where baseline weight is measured at the time of initial administration of DSS or intrarectal TNBS administration. The disease activity index (DAI, 0–12 point scale) was calculated by adding together subscales for percent weight change, stool consistency, and stool blood (fecal occult blood test). Each of the three elements was scored from 0–4 points. The fecal occult blood test was scored as follows: 0 for no color development, 1 for flecks of color reaction, 2 for consistent blue color, 3 for rust-colored stools with a blue reaction, and 4 for wet blood with a dark blue reaction. Stool consistency received a score of 0 for small, firm, dry, nonadherent, and friable stool, 1 for a small firm, moist, and adherent stool, 2 for larger, soft, very adherent stool, 3 for larger, soft, and pliable stool, and 4 for liquid stool. Weight change was also scored from 0 to 4, where % weight loss less than 1% was scored 0, % weight loss between 1% and 5% was scored 1, % weight loss between 5% and 10% was scored 2, % weight loss between 10% and 15% was scored 3, and daily % weight loss greater than 15% was scored 4.

### 4.8. Histological Scoring

A ~1.5 cm piece of the middle of the colon was cut and fixed in 10% phosphate-buffered formalin and then transferred to 70% ethanol. The cassettes were sent to the Translational Pathology Core Laboratory (TPCL) at UCLA for embedding in paraffin, sectioning, and staining with hematoxylin and eosin (H&E). Sections were scored to assess epithelial and mucosal architectural changes as well as the severity and extent of immune cell infiltration using a scoring system from 0–12 with two subscales [57]. For the first subscale, inflammatory cell infiltrates were scored 0–6 based on severity ranging from normal to severe, and the second subscale was based on extent ranging from only the mucosa to transmural involvement. In the first subscore, a score of 1 was assigned for hyperproliferation, irregular crypts, and goblet cell loss, 1.5 for mild crypt loss (10–25%), 2 for moderate crypt loss (25–50%), 2.5 for severe crypt loss (50–75%), 3 for severe crypt loss (75–90%), 4 for complete crypt loss, 5 for ulcers < 10 crypts wide, and 6 for ulcers > 10 crypts wide. For the second subscale, changes in intestinal architecture were scored from 0–6 by adding the scores from each region of the intestinal wall. The mucosa was scored from 0 to 3 (0: normal, 1: mild, 2: modest, 3: severe), the submucosa was scored from 0 to 2 (0: normal, 1: mild to modest, 2: severe), and the mucosa/serosa was scored 0–1 (0: normal, 1: moderate to severe).

### 4.9. Statistical Analyses

Viability data from the cell culture experiment is shown in a bar plot with standard error bars. Significance was determined using the paired nonequal variance *t*-test. Data from histological scoring were shown by violin plots and analyzed by the Mann–Whitney U-test in R (threshold *p*-value < 0.05). Colon length is shown in bar plots, and significance was also assessed by the Mann–Whitney U-test in R. The significance of percent weight loss and DAI scores was determined by linear mixed-effects models using the lme4 package in R.

## Figures and Tables

**Table 1:** Collection period and dominant emission sources for each PM batch in the DSS exposures.

Period	Location	Dominant Emision Source	Collection Period
Campaign I	Los Angeles—sPM	Vehicular emissions	December 2020–January 2021
	Los Angeles—nPM	Vehicular emissions	September 2020–October 2020
Campaign II	Athens PM	Secondary aerosols	August 2020–September 2020
	Milan PM	Residential heating (biomass)	December 2020–January 2021

**Table 2:** Volume and concentration of samples used in cellular assays.

Sample ID	Location	Concentration (μg/mL)	Volume (mL)	Mass (mg)
1	Los Angeles—sPM	65	355	23
2	Los Angeles—nPM	200	115	23
3	Athens PM	200	115	23
4	Milan PM	200	115	23

## Data Availability

Data is contained within the article and Supplementary Material.

## Supplementary Materials

The following supporting information can be downloaded at: https://www.mdpi.com/article/10.3390/ijms25137184/s1.
