# Supplementary material for: Ambient Particulate Matter Induces In Vitro Toxicity to Intestinal Epithelial Cells without Exacerbating Acute Colitis Induced by Dextran Sodium Sulfate or 2,4,6-Trinitrobenzenesulfonic Acid
Source: Int J Mol Sci. 2024 Jun 29;25(13):7184. doi: 10.3390/ijms25137184 (PMC11241079; doi:10.3390/ijms25137184)
Supplement: Supplementary file 1 [file ijms-25-07184-s001.zip › ijms-3035185-supplementary.pdf]

Supplementary

Ambient Particulate Matter Induces In Vitro Toxicity to Intestinal Epithelial Cells without Exacerbating Acute Colitis Induced by Dextran Sodium Sulfate or 2,4,6-Trinitrobenzenesulfonic Acid

Candace Chang [1,2,3,4], Allen Louie [2,3,4], Yi Zhou [1,5], Rajat Gupta [2,3,4], Fengting Liang [1], Georgina Xanthou [1], Jason Ereso [1], Carolina Koletic [1], Julianne Ching Yang [1], Farzaneh Sedighian [1], Venu Lagishetty [1], Nerea Arias-Jayo [1], Abdulmalik Altuwayjiri [6,7], Ramin Tohidi [6,8], Mohamad Navab [2], Srinivasa Tadiparthi Reddy [2,5,9], Constantinos Sioutas [6], Tzung Hsiai [2,10], Jesus A. Araujo [2,3,4,*] and Jonathan P. Jacobs [1,4,11,12,*]

1 Vatche and Tamar Manoukian Division of Digestive Diseases, David Geffen School of Medicine, University of California Los Angeles, Los Angeles, CA 90095, USA; candacechang328@g.ucla.edu (C.C.); zhouyisic@163.com (Y.Z.); fengting.liang@gmail.com (F.L.); gxanthou@bioacademy.gr (G.X.); jasonereso@g.ucla.edu (J.E.); ckoletic@mednet.ucla.edu (C.K.); jcyang1617@g.ucla.edu (J.C.Y.); sedighianfarzaneh@gmail.com (F.S.); vlagishetty@gmail.com (V.L.); nere.arias3@gmail.com (N.A.-J.)

2 Division of Cardiology, David Geffen School of Medicine, University of California Los Angeles, Los Angeles, CA 90095, USA; allenlouie@g.ucla.edu (A.L.); rajatgupta@ucla.edu (R.G.); mnavab@mednet.ucla.edu (M.N.); sreddy@mednet.ucla.edu (S.T.R.); thsiai@mednet.ucla.edu (T.H.)

3 Department of Environmental Health Sciences, Fielding School of Public Health, University of California Los Angeles, Los Angeles, CA 90095, USA

4 Molecular Toxicology Interdepartmental Program, University of California Los Angeles, Los Angeles, CA 90095, USA

5 West China Medical Center, Sichuan University, Chengdu 610017, China

6 USC Viterbi School of Engineering, University of Southern California, Los Angeles, CA 90089, USA; a.altuwayjiri@mu.edu.sa (A.A.); tohidi@usc.edu (R.T.); sioutas@usc.edu (C.S.)

7 Department of Civil and Environmental Engineering, College of Engineering, Majmaah University, Al-Majmaah 11952, Saudi Arabia

8 Air Quality Planning and Science Division, California Air Resources Board, 4001 Iowa Avenue, Riverside, CA 92507, USA

9 Molecular & Medical Pharmacology, University of California Los Angeles, Los Angeles, CA 90095, USA

10 Henry Samueli School of Engineering, University of California Los Angeles, Los Angeles, CA 90095, USA

11 Division of Gastroenterology, Hepatology and Parenteral Nutrition, Veterans Administration Greater Los Angeles Healthcare System, Los Angeles, CA 90073, USA

12 Goodman-Luskin Microbiome Center, University of California Los Angeles, Los Angeles, CA 90095, USA

* Correspondence: jaraujo@mednet.ucla.edu (J.A.A.); jjacobs@mednet.ucla.edu (J.P.J.); Tel.: +1-(310)-825-3222 (J.A.A.); +1-(310)-825-9333 (J.P.J.), Fax: +1-(310)-206-9133 (J.A.A.); +1-(310)-267-1861(J.P.J)

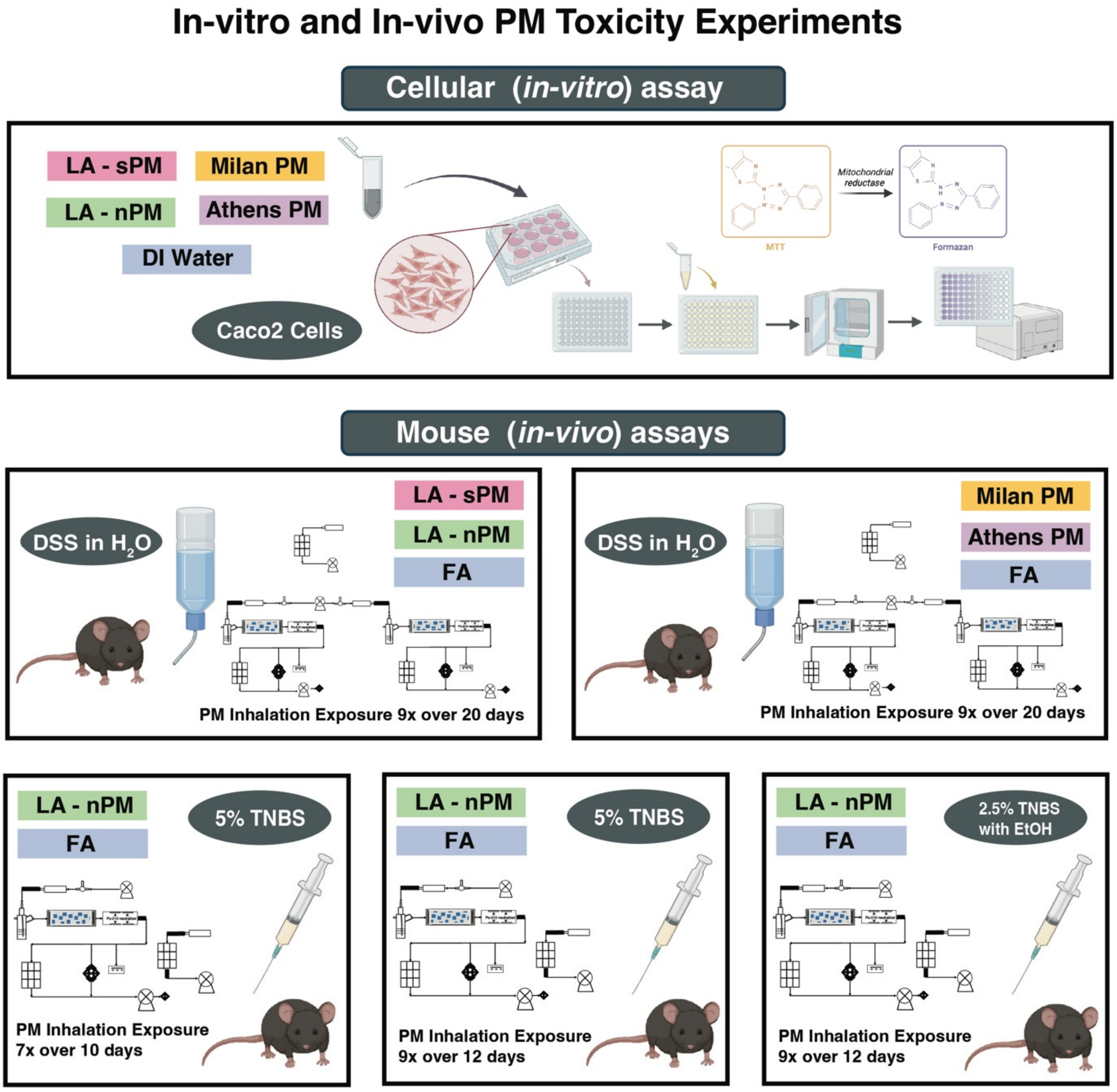

Supplementary Figure S1. Experimental design flowchart. All *in-vivo* experiments used 8 week C57BL/6 male mice. Created with Biorender. Abbreviations: 3-[4,5-dimethylthiazol-2-yl]-2,5 diphenyl tetrazolium bromide (MTT), Los Angeles (LA), Dextran Sodium Sulfate (DSS), Filtered Air (FA), 2,4,6-trinitrobenzenesulfonic acid (TNBS), Particulate Matter (PM), suspended particulate matter (sPM), nano-particulate matter (nPM), De-ionized (DI), Ethanol Alcohol (EtOH), Colon Cancer (Caco).

Supplementary Table S1. Chemical profiles of various PM aerosol used for DSS inhalation exposure experiments.

Composition (µg/mg)

	LA- sPM	LA-nPM	Milan PM	Athens PM
Total carbon	186	119	392	435
Metal elements	86	109	80	195
Water-soluble inorganic ions	206	178	382	360

Selected Metals and Elements (ng/mg)

	LA- sPM	LA-nPM	Milan PM	Athens PM
Ca	12462.87	20980.27	10754.79	31979.41
Al	13462.87	17648.89	5424.49	9856.5
Fe	9886.88	22602.19	5973.15	13183.55
Mg	6222.17	6384.25	4964.33	4888.91
Zn	1550.92	1378.97	618.18	1092.9
Ba	487.30	1155.79	214.18	404.88
Cu	331.28	596.59	241.26	226.55
Ti	774.31	1308.46	119.48	562.11
Mn	283.90	445.07	158.37	301.56
Pb	131.21	235.81	293.95	241.13
Ni	262.81	179.99	37.14	115.26
Sn	48.11	216.82	147.62	135.52
Cr	225.22	345.39	111.43	155.79
V	20.58	40.73	6.6	90.33
Li	12.95	23.93	4.17	8.51
Cd	1.38	4.13	4.84	6.61
Pd	0.80	1.64	4.52	0.56

Water Soluble Inorganic Ions (µg/mg)

	LA- sPM	LA-nPM	Milan PM	Athens PM
Cl	33.2	19.2	9.7	1.1
NO3	105.6	78.8	224.8	3.9
PO4	0.0	1.7	0.8	BDL
SO4	24.8	39.0	40.3	264.2
Na	34.4	27.3	3.0	15.2
NH4	4.7	7.6	91.1	69.6
K	3.5	4.0	11.9	5.8

Supplementary Table S2. Chemical profiles of LA - nPM aerosol used for TNBS inhalation exposure experiments.

Composition (µg/mg)		
	**Cohort 1**	**Cohorts 2&3**
Total carbon	644.34	563.74
Metal elements	106.57	220.99
Water-soluble inorganic ions	319.05	300.41
**Selected Metals and Elements (ng/mg)**		
	**Cohort 1**	**Cohorts 2&3**
Ca	22690.44	22690.44
Al	593.19	593.19
Fe	1363.05	1363.05
Mg	5445.71	5445.71
Zn	1316.05	1316.05
Ba	330.63	330.63
Cu	470.02	470.02
Ti	115.07	115.07
Mn	115.24	115.24
Pb	34.36	34.36
Ni	408.43	408.43
Sn	30.15	30.15
Cr	244.73	244.73
V	11.07	11.07
Li	73.91	73.91
Cd	3.78	3.78
Pd	0.36	0.36
**Water Soluble Inorganic Ions (µg/mg)**		
	**Cohort 1**	**Cohorts 2&3**
Cl	26.59	13.27
NO3	50.27	52.5
PO4	BDL	BDL
SO4	182.96	159.29
Na	50.3	61.41
NH4	BDL	3.43
K	8.9	10.5
